# Respiratory Activity Classification Based on Ballistocardiogram Analysis

**DOI:** 10.1007/978-3-030-51517-1_7

**Published:** 2020-05-31

**Authors:** Mohamed Chiheb Ben Nasr, Sofia Ben Jebara, Samuel Otis, Bessam Abdulrazak, Neila Mezghani

**Affiliations:** 8 Digital Research Centre of Sfax, Sfax, Tunisia; 9 Institut Mines-Télécom, CNRS, Paris, France; 10 Université de Sherbrooke, Sherbrooke, QC Canada; 11 Digital Research Centre of Sfax, Sfax, Tunisia; 12 University of Sfax, Sfax, Tunisia; 13 Higher School of Communication of Tunis, Carthage University, Aryanah, Tunisia; 14 Laboratoire de recherche en imagerie et en orthopédie, CRCHUM, Montreal, Canada; 15 LICEF Institute, TELUQ University, Montreal, Canada; 16 Department of Computer Science, Sherbrooke University, Sherbrooke, Canada

**Keywords:** Ballistocardiogram, Machine learning, Biomedical signal processing, Spectral analysis

## Abstract

Ballistocardiogram signals describe the mechanical activity of the heart. It can be measured by an intelligent mattress in a totally unobtrusive way during periods of rest in bed or sitting on a chair. The BCG signals are highly vulnerable to artefacts such as noise and movement making useful information like respiratory activities difficult to extract. The purpose of this study is to investigate a classification method to distinguish between seven types of respiratory activities such as normal breathing, cough and hold breath. We propose a feature selection method based on a spectral analysis namely spectral flatness measure (SFM) and spectral centroid (SC). The classification is carried out using the nearest neighbor classifier. The proposed method is able to discriminate between the seven classes with the accuracy of 94% which shows its usefulness in context of Telemedicine.

## Introduction

The development of connected object for personalized services, especially for monitoring purposes, have significantly increased worldwide over the last few years [1]. More specifically those that deals with the monitoring of respiratory and cardiac diseases. Indeed these diseases are among the leading cause of death and disability in the world. One of these respiratory diseases is the Chronic Obstructive Pulmonary Disease COPD [2] a progressive life threatening lung disease. According to the World Health Organization [3], COPD affects more than 250 million cases globally, a staggering 3.17 million deaths per year and is associated with a huge economic burden. In fact, numbers published by the Global initiative for Chronic Obstructive Lung Disease [4] shows that the direct costs of respiratory disease in the European Union are estimated to be about $6\%$ of the total annual healthcare budget with COPD accounting for $56\%$ (38.6 billion Euros) of the cost of respiratory disease. These numbers are further amplified by the ever-growing healthcare costs, the aging of the population and the widespread of such diseases. The monitoring of respiratory activities plays an important role in the current management of patients with acute respiratory failure [5]. As a consequence, it is recommended to have continuous monitoring of the vital signs to ensure an optimal diagnosis of a patient’s state [6]. Moreover, monitoring of respiratory activity is useful for detecting respiratory disorders, such as the sleep apnea, cessation of breathing in infants, shortness of breath in patients with heart failure, and so on. Hence, it is important to monitor respiratory activities such as normal breathing, cough, hold breath expiration.

A new generation of sensor-based mattress is able to unobtrusively monitor vital signs such as the Heart rate Beat Rate (HBR) and the Respiratory Rate (RR). Indeed, this study considered an Optical Fiber based Sensor (FOS) [7] for the unobstructed monitoring of the Ballistocardiogram (BCG) signal. Due to the ejection of the blood during the systole, the body’s mechanical reaction is measured hence the BCG signal. Our aim is to investigate a classification method to distinguish between several types of respiratory activities such as normal breathing, cough and hold breath using the BCG signal.

This paper is organized as follows. Section 2 is dedicated to describe the material and method. It describes the data collection, BCG signal analysis and feature extraction and classification. Section 3 provides information about the experimental results mainly feature illustration and classifier evaluation. Finally, Sect. 4 concludes the study and gives perspectives.

## Material and Method

### Data Collection

The system used for collecting data includes a small FOS mattress and a module to gather optical data coming from the mattress [8, 9]. The FOS mattress was fixed on the back of a regular office chair. The raw data is sampled at 50 Hz by the module.

**Fig. 1.**
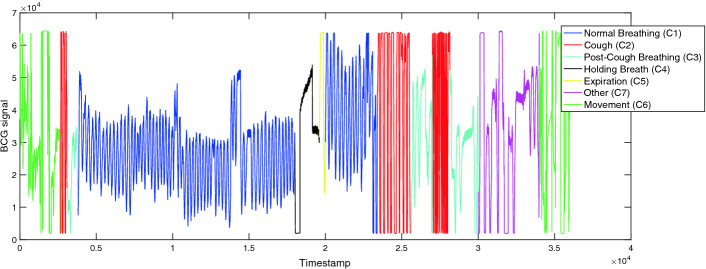
Illustration of the BCG signal during the experimental protocol activities. (Color figure online)

The BCG signals were acquired on 6 healthy participants: 3 male and 3 female aged between 21 and 32 years. The participants were asked to perform a certain experimental protocol. A part of normal breathing, other human body activities that commonly occur are introduced in this protocol. It is composed of the following steps by following activities: normal breathing (C1), cough (C2), Normal breathing after cough (C3), hold breath (C4), expiration (C5), movement (C6). We also consider a class other to regroup all other activities (C7). Figure 1 illustrates an example of the BCG signal. The different human body activities are plotted in different colors. The objective is to highlight the differences in the BCG signal according to the activity.

### BCG Signal Analysis

**Fig. 2.**
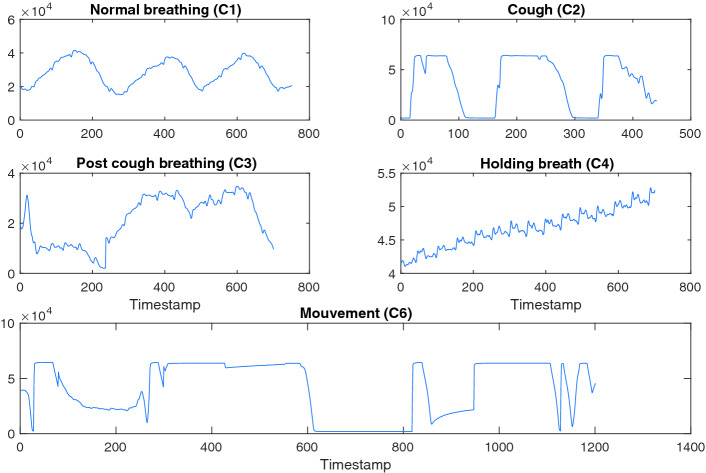
Illustration of the BCG signal of the different activities of the subjects.

In this subsection we inspect the effect of different activities in the BCG signal. Figure 2 illustrates five different activities. In the plot illustrating Normal respiration (C1), we can easily extract both the HBR and RR, the big period corresponds to the movements of the thoracic cage. By extracting the distance between two consecutive peaks of this waveform, we can extract the RR. The small period appearing in the BCG signal represents the heart beats. The extraction of these little fluctuations results in the extraction of the HBR.

The signals corresponding to the cough (C2) and movement (C6) are very similar. Both signals attain the upper limit of the acquisition equipment which is explained by the broad peaks in the BCG signals. We believe however that these broad peaks have different explanations. The peaks in the movement signal comes from the acceleration of the subject’s body and the peaks during the cough comes from the reaction of the body after coughing.

The post-cough normal breathing is corrupted and we can hardly find the peaks of the respiratory activity. The peaks are broader and that results in a lack of precision of HBR and RR.

The holding breath BCG contains only cardiac information. The periodicity is clearly noticed which was not obvious in other activities.

This analysis of the BCG signal’s content motivates the use of an approach based on the characterization of the useful frames. This particular problem is complex and thus is demanding when it comes to the choice of the features with physical significance. In the next subsections, we will define the features and try to highlight the intuition behind each one of them.

### BCG Signal Feature Extraction

A periodic signal can be represented as a sum of sine waves and thus the Fourier transformation of this particular signal will be spiky. This statement motivated the idea of using the following two features: Spectral Flatness Measure (*SFM*) and Spectral Centroid (*SC*).

Let *x*(*n*) be a BCG signal. The later is decomposed into frames of short duration. These frames should be long enough to carry information about the activity but not too long to avoid an overlap of two or more different activities. In the frequency domain, the short-term Fourier transform is calculated and its amplitude is extracted. It is denoted |*X*(*m*, *k*)|, where *m* is the frame index and *k* is the discrete frequency.

**Spectral Flatness Measure (SFM):** The SFM, also known as Wiener entropy, is a signal processing measure used to describe the flatness of the spectrum of the signal [10, 11]. The *SFM* is defined as the ratio of the geometric mean and arithmetic mean of the Fourier transforms. When the spectrum is flat (white noise signal), the resulting measure is close to 1.

1 $$\begin{aligned} SFM(m)=\frac{\root N \of {\prod _{k=1}^N |X(m,k)|}}{\frac{\sum _{k=1}^N |X(m,k)|}{N}}, \end{aligned}$$

where *k* is the frequency bin index and *N* is the number of frequency bins.

**Spectral Centroid (SC):** The SC indicates where the center of mass of the spectrum is located.

2 $$\begin{aligned} SC(m)=\frac{\sum _{k=1}^N(|X(m,k)|*f(k))}{\sum _{k=1}^N |X(m,k)|}, \end{aligned}$$

where *f*(*k*) is the frequency in Hz of the bin *k*.

**BCG Feature Engineering:** The SFM and SC measures have been evaluated on each frame of the BCG signal. However, in order to avoid complexity that comes with it (overlap noise propagation, presence of different labels in the same frame, frames too small to be representative...), we propose to create a time-series out of the SFM and SC values in each frame.

- Signal decomposition: the original BCG signal is decomposed into frames of length 1024 with an overlap of 960 samples. Hence we used a windowing function. In this work we used a *Hamming* window with an increment of 64 samples.
- The feature vector $F(m)=[SFM(m), SC(m)]^T$ is extracted from each frame.
- Each feature of raw data $SFM=[sfm(1),sfm(2)...sfm(L)]^T$ (*L* is the number of frames) is transformed into a time-series (equivalent to a signal) by overlapping and adding the *sfm* of each frame. Note that the latter is a constant vector, whose value is *sfm* and whose length is the frame size.

The $sfm_{signal}$ and $sc_{signal}$ are then used for the purpose of classification.

### Activities Classification

Respiratory activities classification has been performed using a K-nearest neighbors classifier. It is a non-parametric classification method which classifies a sample based on a plurality vote of its neighbors. The sample is assigned to the class most common among its *K* nearest neighbors (*K* is a positive integer) in terms of minimal distance. The algorithm adopted is Fine *KNN* which is the finest variation of *KNN* since it labels the new input with the same label as only one of its nearest neighbour $K=1$. The evaluation of the algorithm as well as the classification results were conducted using k-fold cross validation with $k=5$.

### Classification Evaluation

The classification performance is evaluated in terms of true positive rate and positive predicted value.

**True Positive Rate:** The performance of our model will mainly be measured using the confusion matrix [12]. Specifically *TPR* measures the proportion of detected positives from the actual positive in other terms *TPR* measures how sensitive your model is to the positive class.

3 $$\begin{aligned} TPR^{~i}=\frac{true~positives}{true~positives+false~negatives}, \end{aligned}$$

where *i* corresponds to the class (activity) of the subject (*i* = 1..7). The terms of the confusion matrix presented in Fig. 4 are defined as follows:

4 $$\begin{aligned} Conf_{tpr}(i,j)=\frac{M_{ij}}{\sum _{j=1}^C M_{ij}}, \end{aligned}$$

where *C* is the number of classes, $M_{ij}$ is the number of predictions of class i that actually belongs to class j it is usually measured by comparing the test results to the ground truth.

**Positive Predictive Value:** The proportion of the predictions made that are actually true and happened. *PPV* Highlights mostly how refined our model is and how frequent we have false alerts.

5 $$\begin{aligned} PPV^{~i}=\frac{true~positives}{true~positives+false~positives}. \end{aligned}$$

The terms of the confusion matrix presented in Fig. 5 are defined as follows:

6 $$\begin{aligned} Conf_{ppv}(i,j)=\frac{M_{ij}}{\sum _{i=1}^C M_{ij}}, \end{aligned}$$

## Experimental Results

### Feature Illustration

This section illustrates the feature analysis and interpretation by providing the means of the *SFM* and *SC* for each activity.

The BCG signal we are working with is the same displayed on Fig. 1. The mean values are given in Table 1. We note that the normal breathing mean value of the *SFM* is the lowest which confirms the periodicity hypothesis. The values of *SFM* and *SC* taken during the coughing portion (C2) as well as the movement portion (C6) are relatively high which further confirms the non-periodicity in the corresponding portions.

For the post-cough breathing, we notice that, unlike the portion of normal breathing, the values of the descriptors are high and close to those during the movement and coughing activities which supports our choice to isolate these portions.

The closest values of the descriptors to the ideal ones (those of the normal breathing) are the values recorded during the holding breath, this is due to the fact that the portions of holding breath are periodic and carry only the heart rhythm information.

**Table 1.** Mean value of *SFM* and *SC* during each activity.

Activities	*SFM*	*SC*
Normal respiration (C1)	0.02	0.807
Cough (C2)	0.1582	2.3591
Post cough breathing (C3)	0.14	1.2638
Holding breath (C4)	0.102	0.7662
Movement	0.2293	1.4844

**Fig. 3.**
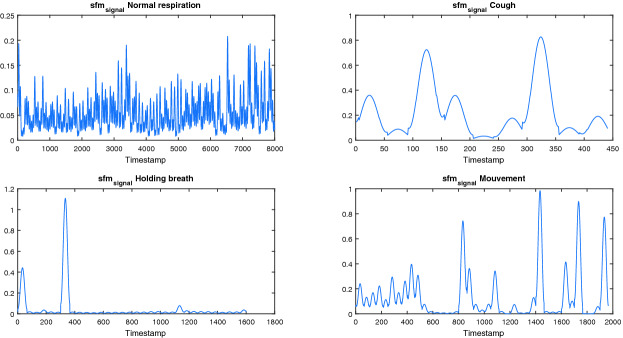
Variation of sfm signal of the sample for each activity.

Figure 3 show the $sfm_{signal}$ of some activities. In the top right, the normal respiration phase is considered. The values of *SFM* are low as expected to be. This fact is due to the clear periodicity in that activity. The *SFM* of holding breath (down left plot) is pretty low and that is as well an expected result since there’s the cardiac information in the holding breath activity. Both cough and movement activities (right plots) manifest big fluctuations in their respective $sfm_{signal}$, this is due to the absence of periodicity in these signals.

### Classifier Evaluation

**Fig. 4.**
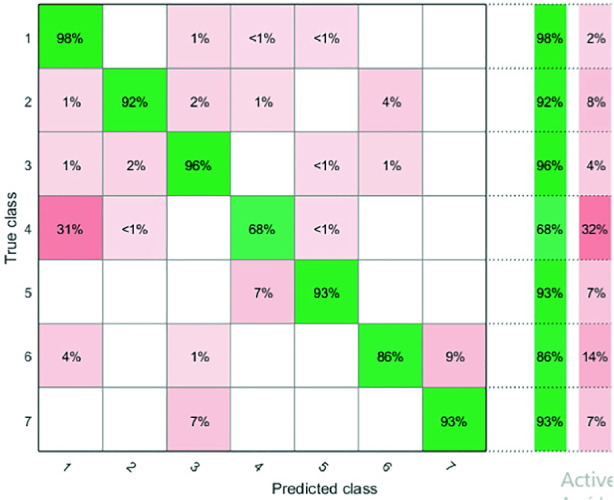
*TPR* confusion matrix

Using the $sfm_{signal}$ and $sc_{signal}$ we obtained a classification rate of 94%. Figure 4 shows the TPR confusion matrix. We can observe that our model performs very well overall. The *TPR* value appears in the diagonal of the confusion matrix. Most of the errors are detected in the class *C*4, where $31\%$ of the latter (corresponds to the class: holding breath) is predicted as normal respiration. This result is expected since the periodicity in the holding breath portions is present due to the cardiac activity. We observe as well a high confusion between the class cough (C2) and the class movement (C6). This is due to the fact that our predictors are well equipped to detect the existence of the periodicity in the portions.

**Fig. 5.**
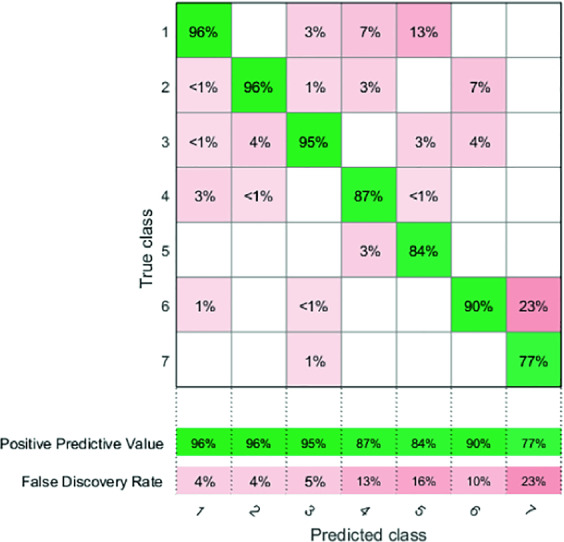
*PPV* Confusion matrix

Figure 5 shows the PPV confusion matrix. We can observe the confusion between the classes C1 and C4 ($7\%$) in terms of PPV this corresponds to a high false-alert rate (the majority of false alerts in the class: Normal respiration are recorded as Holding breath) which further confirms the similar periodicity hypothesis mentioned in the previous paragraph we can also pinpoint an alarming $13\%$ with the class C5 which is due to the lack of the adopted features when it comes to differentiating between the highly non-periodic portions.

## Conclusion

In this study we investigated the respiratory activity classification based on the BCG signal. We used a reconstructed time-series signal from the spectral flatness measure (SFM) and spectral centroid (SC) of the raw data. We obtained a classification rate of 94% which show the effectiveness of the proposed method. The supervised classification process, however, is demanding when it comes to data and computation. Treating the feature extraction process by generating a time series is a novelty which motivates the use of more sophisticated deep-learning algorithms such as the Long Short Term Memory LSTM as one of the most used Recurrent Neural Network RNN architectures in time series related problems.

## References

[1] Wearable Monitoring System for Chronic Cardio-Respiratory Diseases. In: 30th Annual International IEEE EMBS Conference Vancouver, British Columbia, Canada, 20–24 August 2008 10.1109/IEMBS.2008.4650010 19163513

[2] Forum of International Respiratory Societies (2017). The Global Impact of Respiratory Disease.

[3] Who.int. Chronic respiratory diseases (2020). https://www.who.int/health-topics/chronic-respiratory-diseases. Accessed 18 Feb 2020

[4] Global strategy for the diagnosis, Management and prevention of Chronic Obstructive Pulmonary Disease 2020 report

[5] Clinical review: Respiratory monitoring in the ICU - a consensus of 16. Crit Care. **16**(2), 219 (2012). 10.1186/cc11146. PMCID: PMC3681336PMID: 22546221 10.1186/cc11146 PMC3681336 22546221

[6] Liu, J., Wang, Y., Chen, Y., Yang, J., Chen, X., Cheng, J.: Tracking vital signs during sleep leveraging off-the-shelf WiFi. In: Proceedings of the 16th ACM International Symposium on Mobile Ad Hoc Networking and Computing, MobiHoc 2015, New York, NY, USA, pp. 267–276. ACM (2015)

[7] Sadek I, Biswas J, Abdulrazak B (2019). Ballistocardiogram signal processing: a review. Health Inf. Sci. Syst..

[8] Ramakrishnan M, Rajan G, Semenova Y, Farrell G (2016). Overview of fiber optic sensor technologies for strain/temperature sensing applications in composite materials. Sensors.

[9] Otis, S., Mezghani, N., Abdulrazak, B.: Comparative Study of Heart Rate Extraction Methods for a Novel Intelligent Mattress. In: IEEE International Symposium on signal Image, Video and Communications (ISIVC), pp. 27–29, Rabat, Morocco (2018)

[10] Dubnov S (2004). Generalization of spectral flatness measure for non-gaussian linear processes. IEEE Sig. Process. Lett..

[11] Madhu N (2009). Note on measures for spectral flatness. Electron. Lett..

[12] Stehman S (1997). Selecting and interpreting measures of thematic classification accuracy. Remote Sens. Environ..

